# Supplementary material for: Immune responses in COVID-19 respiratory tract and blood reveal mechanisms of disease severity
Source: Res Sq. 2021 Aug 26:rs.3.rs-802084. Preprint. [Version 1] doi: 10.21203/rs.3.rs-802084/v1 (PMC8404907; doi:10.21203/rs.3.rs-802084/v1)
Supplement: Supplement 1 [file 4ae24813dc48b05365e1a81b.pdf]

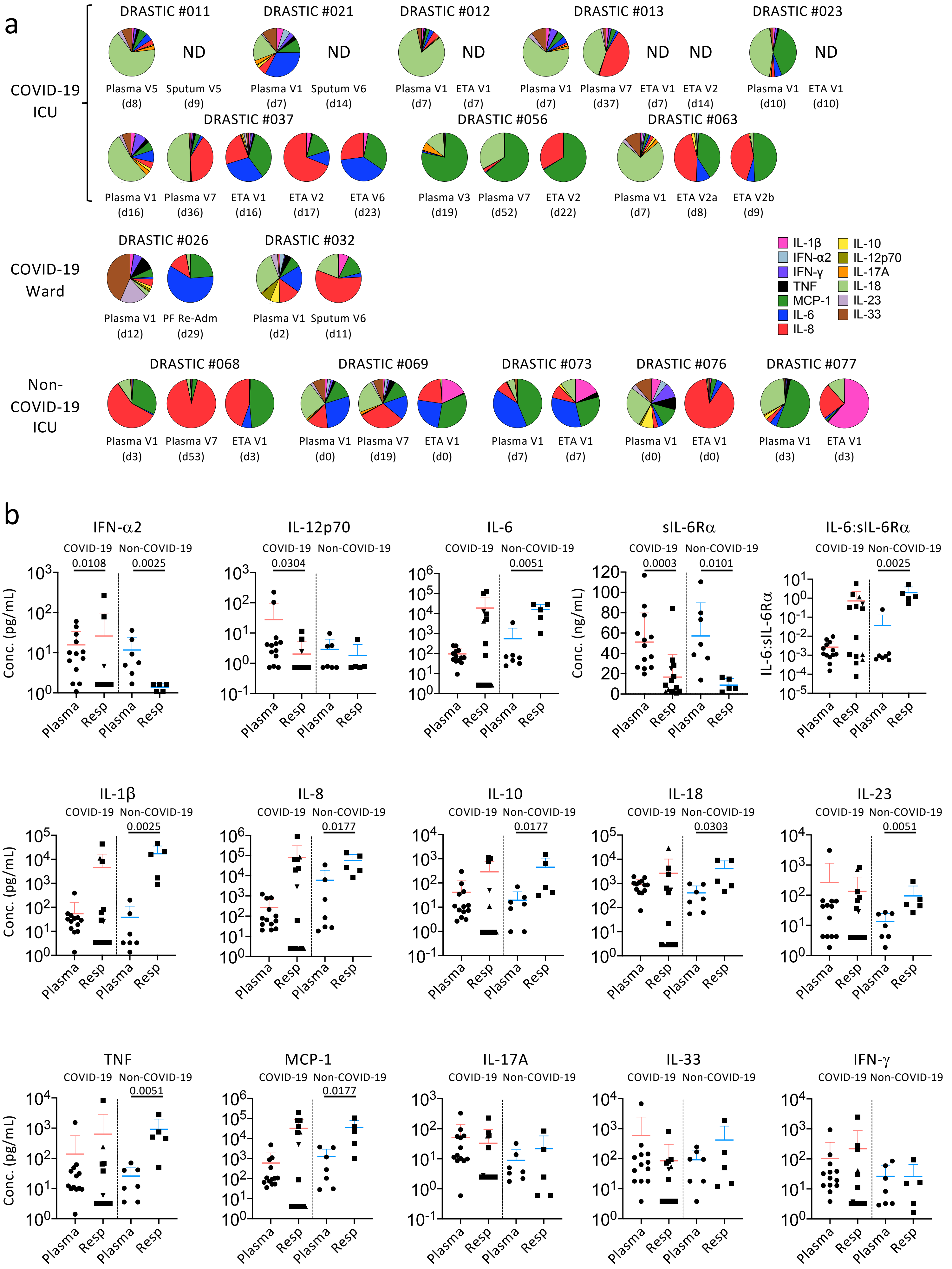

Supplementary Fig. 1 Zhang *et al*

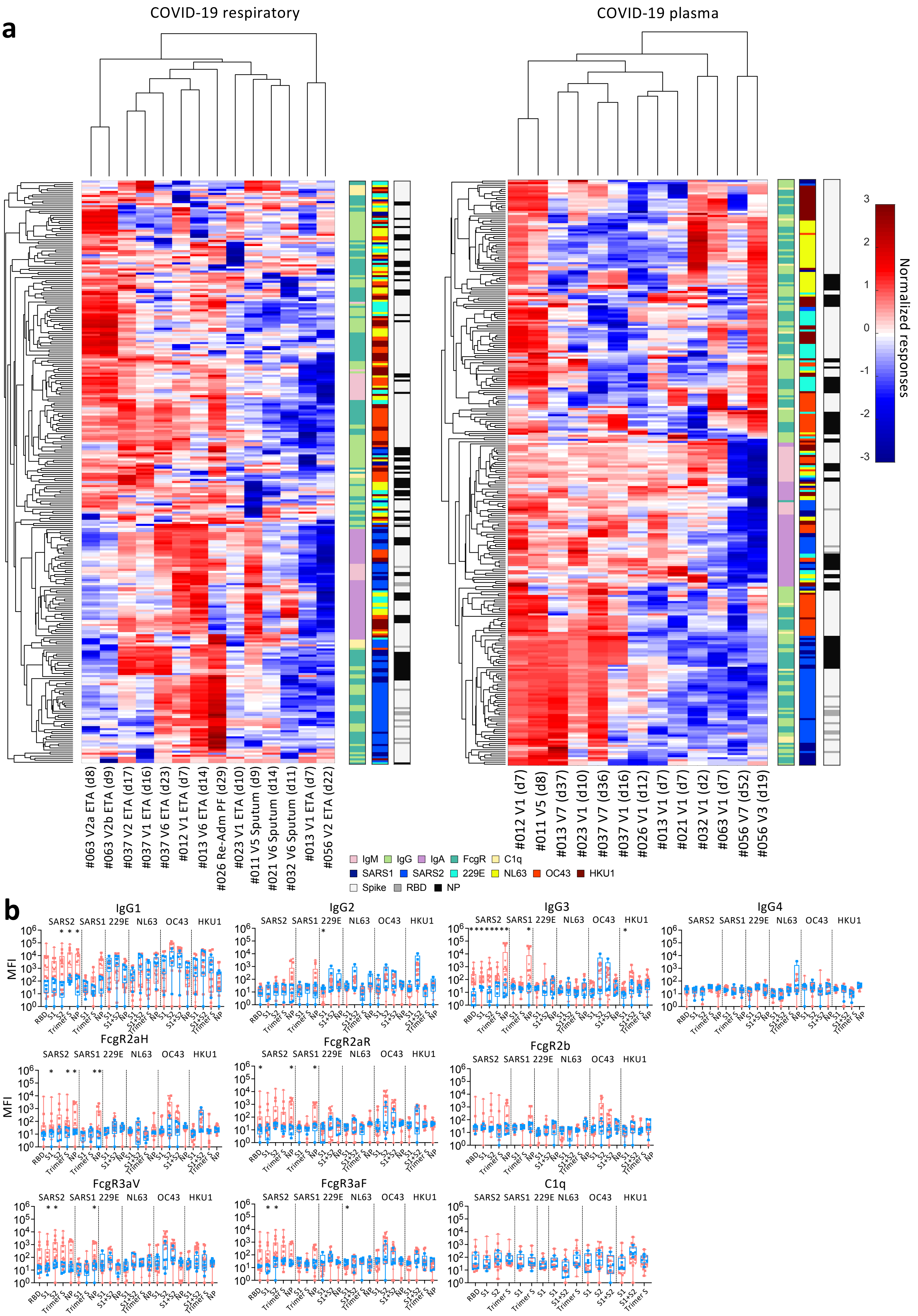

Supplementary Fig. 2 Zhang *et al*

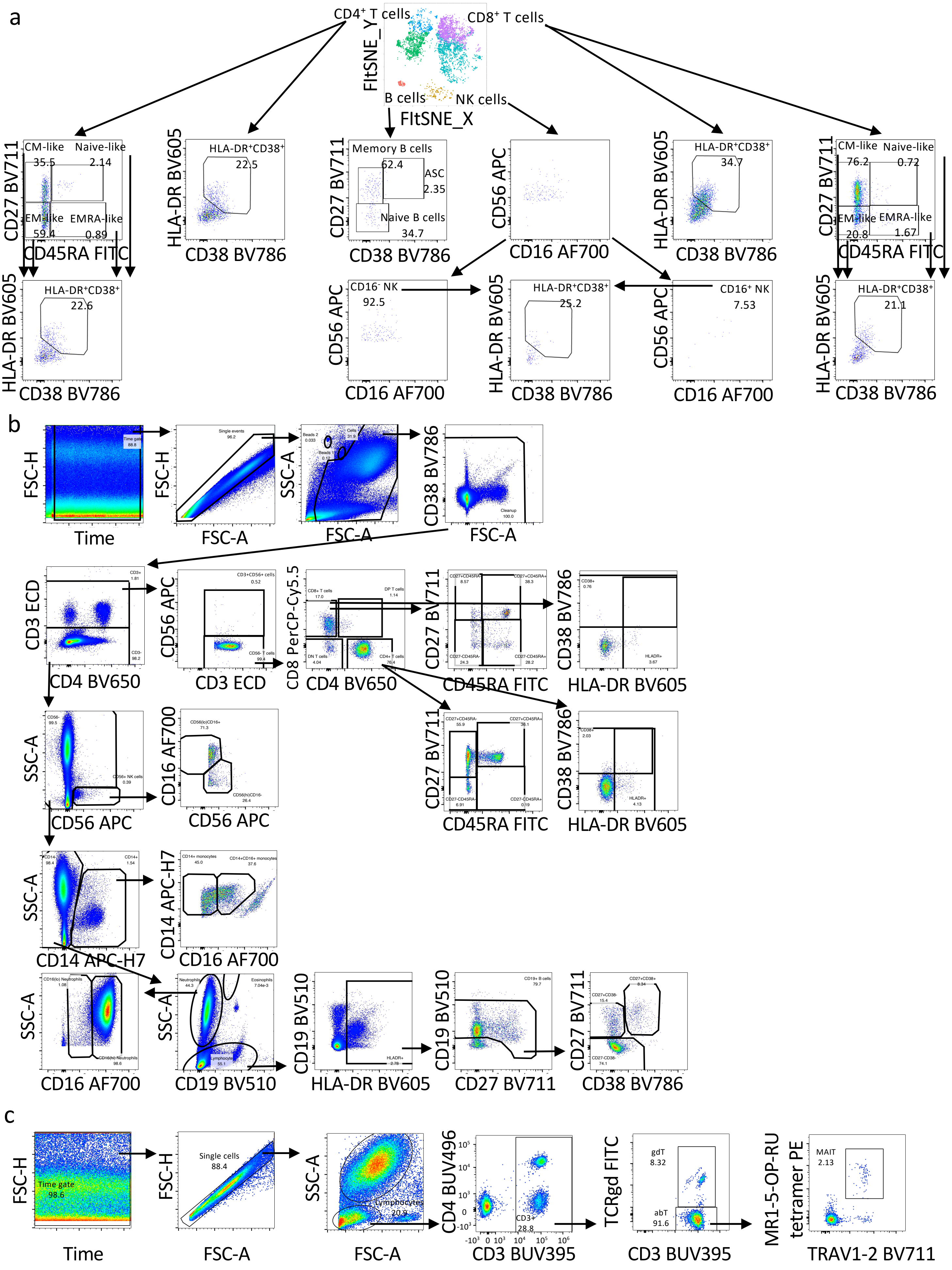

Supplementary Fig. 3 Zhang *et al*

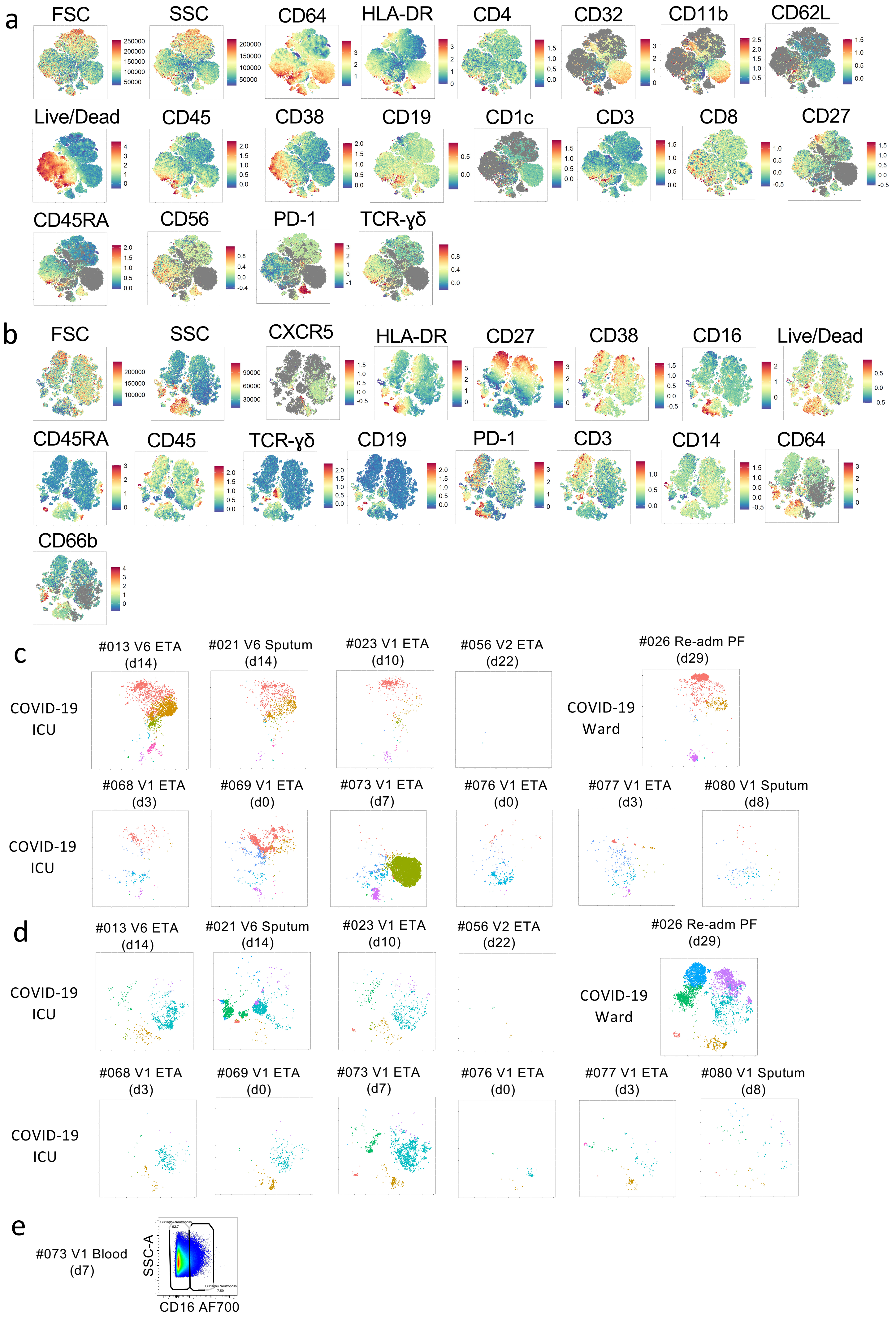

Supplementary Fig. 4 Zhang *et al*
